# Retina images classification based on 2D empirical mode decomposition and multifractal analysis

**DOI:** 10.1016/j.heliyon.2024.e27391

**Published:** 2024-03-04

**Authors:** Lei Yang, Minxuan Zhang, Jing Cheng, Tiegang Zhang, Feng Lu

**Affiliations:** a School of Mechatronic Engineering and Automation, Shanghai University, China; b College of Electrical Engineering, Sichuan University, China

**Keywords:** Texture analysis, Multifractal spectra, Hilbert-Huang transform, 2D-EMD, Retina

## Abstract

Diabetic retinopathy is an ocular disease caused by long-term damage to the retina due to high blood sugar levels. Elevated blood sugar can impair the microvasculature in the retina, leading to vascular abnormalities and the formation of abnormal new blood vessels. These changes can manifest in the retina as hemorrhages, leaks, vessel dilation, retinal edema, and retinal detachment. The retinas of individuals with diabetes exhibit different morphologies compared to those without the condition. Most histological images cannot be accurately described using traditional geometric shapes or methods. Therefore, this study aims to evaluate and classify the morphology of retinas with varying degrees of severity using multifractal geometry. In the initial experiments, two-dimensional empirical mode decomposition was employed to extract high-frequency detailed features, and the classification process was based on the most relevant features in the multifractal spectrum associated with disease factors. To eliminate less significant features, the random forest algorithm was utilized. The proposed method achieved an accuracy of 96%, sensitivity of 96%, and specificity of 95%.

## 1 Introduction

The impact of diabetes on vision is primarily associated with diabetic retinopathy, where high blood sugar adversely affects retinal cells and microvessels, leading to the occurrence of diabetic retinopathy [1]. At first, diabetic retinopathy might cause no symptoms or only mild vision problems. But it can lead to blindness. Many research studies have been conducted in this field. In Thakur's study [2], the author provides an overview of how various researchers have summarized segmentation techniques for the optic disc and cup, with a particular emphasis on their application in the diagnosis of glaucoma. A comprehensive assessment was conducted in Ref. [3], which delved into registration methods and automated detection techniques for color fundus images in diabetic retinopathy. In Ref. [4], an improved deep forest model, called MFgcForest (Multi-class feature Extraction deep forest), for multi-classification of diabetic retinas has been proposed. In Ref. [5], a new approach that involves capturing the retinal fractals from images and analyzing these derived retinal fractals to assist in disease prediction was approached.

Although multifractals have been used for texture analysis applications involving natural images, their capability to characterize the intensity of textures has not been fully exploited, especially in the field of medical imaging. Fractal theory is initially a branch of mathematics that is built upon set theory, function theory, and measure theory. By analyzing the Hausdorff measure of fractal objects (including fractal sets, fractal functions, etc.), it extends the concept of integer dimension in Euclidean geometry to fractional dimensions. Fractal dimension remains unchanged under bi-Lipschitz transformations, which ensures its resilience against geometric distortions. As a result, fractal analysis provides an efficient approach for describing the textures and structures present in medical and natural images processing.

Fractal dimension of cerebellar regions were estimated from magnetic resonance images of patients with CM-I and healthy subjects in Ref. [6]. In Ref. [7], the 2D Wavelet-Transform Modulus Maxima method was used to detect microcalcifications in human breast tissue seen in mammograms and to characterize the fractal geometry of benign and malignant MC clusters. The proposed work utilizes multifractals to characterize the trabecular bone texture in the radiographs in Ref. [8]. Authors firstly calculate the Holder exponent to determine the Hausdorff dimension and quantify the global regularity of pixels. Finally, the porosity was calculated based on the Hausdorff dimension. Fractal dimension is utilized in Ref. [9] to analyze panoramic radiography and nuclear medicine scans. The methodology involves initially quantifying the intensity differences between pixels at various scales, followed by estimating the fractal dimension through the slope calculation of the logarithmic plot of intensity differences against scale. In Ref. [10], authors use multifractal analysis to assess the effectiveness of guided bone regeneration in the healing process of post-extraction and post-crystallization bone loss. In Ref. [11], a textural analysis method based on fractals is presented, which employs intermittent multiscale fractional Brownian motion to characterize the textures and classify corn silage images. The authors present a texture classification system in Ref. [12], which is based on a learning approach and does not rely on any pre-existing models. Additionally, the author explores the problem of image segmentation and introduces the concept of mixed classes. This concept allows for precise detection of texture boundaries in complex images.

In Ref. [13], authors propose a novel approach for the detection and classification of retina images using a hybrid system that combines two-dimensional multifractal detrended fluctuation analysis and least square support vector machines. In Ref. [14], authors propose a novel approach to analyze the complexity of the retinal vascular system by extracting the Fourier fractal dimension at different wavelet scales to enhance its visibility. The fractal dimension of the retinal vascular system in high-resolution fundus images is quantified using multifractal analysis in Ref. [15]. Another method in Ref. [16] utilizes Gabor wavelet transform for preprocessing the green channel of the retinal image. The authors of the study conducted a pairwise Pearson correlation analysis between the estimated fractal dimensions of each of the 380 images and 100 simulated regions of interest.

Concerning the classification of pathological retinal images, the extraction of distinctive and representative features emerges as a critical factor for achieving favorable classification results. We found that the distribution of blood vessels in the retina exhibits self-similar characteristics, making it highly suitable for applying multifractal methods to analyze the texture of the vessel distribution. Different variants of multifractal analysis, such as wavelet leader-based multifractal analysis, detrended fluctuation analysis, and wavelet transform modulus maxima-based methods have been utilized in relevant fields. However, there have been limited experiments combining multifractal analysis with two-dimensional Hilbert Huang transform. The Hilbert Huang Transform is a data analysis method that combines the Hilbert Transform and the Empirical Mode Decomposition to analyze signals or time series data. Compared to theory and algorithms, HHT has achieved faster development in terms of applications and has been widely adopted by scholars from various countries in various fields within a short span of a few years. In the field of biomedical research, EMD can be used for pulse wave signal analysis, brainwave analysis, estimation of resonant frequencies in speech signals, etc. In seismology, it is used for analyzing seismic wave signals. In structural analysis, it is used for structural damage detection, bridge safety inspection, etc.

In Ref [17], the authors use Hilbert Huang transform to characterize the instability of rock fractures in terms of time domain-frequency domain-energy deposition. In Ref. [18], an enhanced approach that combines Hilbert-Huang Transform with Wavelet Packet Transform is proposed for the recognition of continuous electroencephalogram signals in Brain-Computer Interfaces. In Ref. [19], a novel logarithmic frequency representation of the signal derived using the HHT is proposed. In Ref. [20], Hilbert-Huang transform is used to enhance the accurate delivery and assure efficient recognition of power quality events in the electrical systems. In Ref. [21], a fault diagnostic method for a distribution grid that consists of the Hilbert-Huang transform and feedforward neural networks is presented. Many experiments have applied Hilbert-Huang Transform to one-dimensional signal processing, but there are few examples of its application on two-dimensional images. This paper extends it to the two-dimensional domain and applies it to medical image processing. The images' various frequency components are extracted and studied individually.

## 2 Methodology

### 2.1 Outline

In the proposed method, the first step is to perform a two-dimensional Hilbert transform on the retinal images. We believe that in dealing with non-linear and non-stationary signals, the Hilbert-Huang transform is a method superior to the short-time Fourier transform and wavelet transform. Traditional methods such as Fourier transform and wavelet transform can only analyze linear stationary signals, while Hilbert Huang transform can analyze nonlinear non-stationary signals. This makes it more flexible and effective in dealing with practical problems. Compared to methods such as wavelet transform, Hilbert Huang transform can provide higher resolution in the frequency domain. Because it uses a method based on local frequency distribution to describe the spectral characteristics of signals, it has advantages in dealing with problems that require high-precision frequency domain analysis. The Hilbert Huang transform does not require pre-defined templates or rules, making it easy to extend to new signal types and domains. This makes it more flexible and useful in practical applications.

**Fig. 1:**
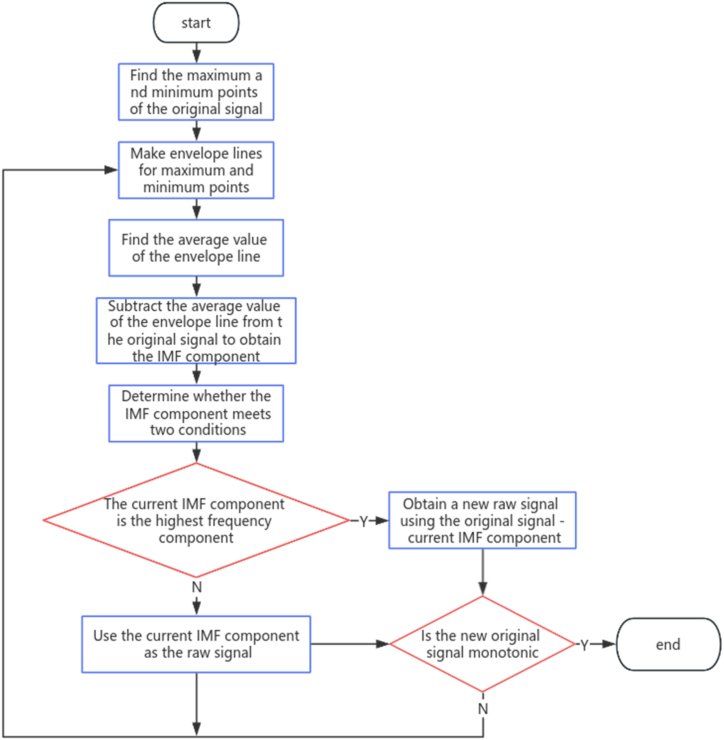
The workflow of the EMD.

The prerequisites for Hilbert transform include that the signal is real, has a finite duration, is stationary and monotonic. Only when these prerequisites are met can the Hilbert transform be effectively applied for signal processing. Because most signals in reality have no regularity and do not meet the usage requirements, it is necessary to perform Hilbert transform on the intrinsic mode function (IMF) obtained from empirical mode decomposition (EMD) to obtain complex signals before further analysis. Fig. 1 is a general outline of the EMD (Empirical Mode Decomposition) method.

In the second step, we perform multifractal analysis on the images after the Hilbert-Huang transform. It is a tool used to describe the fractal properties of complex systems at multiple scales. It reveals the distribution and variations of the fractal nature of the system at different scales by measuring and analyzing the local fractal dimensions of complex systems. Next, we separately generate multifractal spectra for the different components obtained from the first part of the processing, selecting suitable components as subsequent classification indicators. Through experimental verification, we discovered that one component exhibits more prominent fractal characteristics compared to the other components. Based on this, we chose to generate a multifractal spectrum for this component and examine the distribution of fractal spectrum widths.

**Fig. 2:**
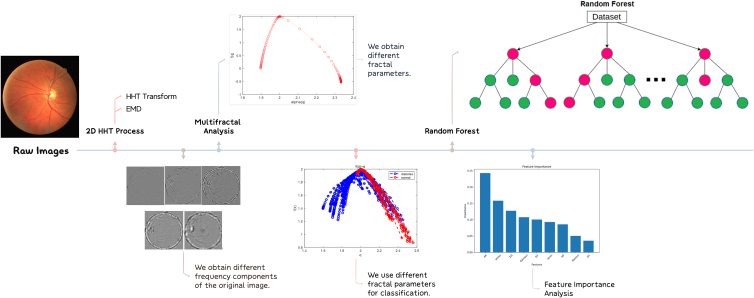
The workflow of the proposed methodology.

In the thrid step, we use the parameters obtained from the multifractal processing as indicators to perform k-means clustering on retinas from different populations and conduct Spearman correlation analysis on these parameters. To eliminate the less important features, the Random Forest algorithm has been used. The workflow of the proposed methodology is illustrated Fig. 2.

### 2.2 Hilbert-Huang Transform and its 2D extension

Traditional analysis methods, such as Fourier transform, have limitations in handling such signals and cannot provide accurate results. Therefore, in order to overcome these limitations, the Hilbert-Huang Transform (HHT) was introduced. The HHT is an adaptive time-frequency analysis method [22] that was proposed by N.E. Huang et al., in 1998. The main purpose of this method is to analyze nonlinear and non-stationary signals. The Hilbert-Huang transform consists primarily of two components: Empirical Mode Decomposition (EMD) and Hilbert Spectral Analysis (HSA) [23]. Firstly, the signal is decomposed through Empirical Mode Decomposition, which involves breaking down the signal into a set of intrinsic mode functions (IMFs) [24]. Subsequently, the Hilbert transform is applied to each IMF to obtain instantaneous frequency information.

The 2D Hilbert-Huang Transform [25] is a signal processing technique used for handling two-dimensional data, such as images or image sequences. It is an extension of the one-dimensional Hilbert-Huang Transform, designed to extract local features, mode components, and oscillatory patterns from two-dimensional data [26]. This method combines Empirical Mode Decomposition and Hilbert Transform to analyze and extract patterns and information from the data. By applying the 2D Hilbert-Huang Transform, it is possible to decompose and analyze two-dimensional data in various scales and orientations, making it valuable for applications in fields such as image processing, signal processing, and pattern recognition.

Regarding the uniqueness of texture signals in images, extending one-dimensional Empirical Mode Decomposition to two-dimensional Empirical Mode Decomposition poses several challenges, including the selection of extrema points on the two-dimensional surface, constructing envelope surfaces for fitting, defining stopping criteria, and addressing boundary issues.

In this paper, we extend the one-dimensional SD stopping criterion to the two-dimensional empirical mode decomposition. The sifting stopping criterion SD for the i-th intrinsic mode function component is as follows [27]:

(1) $$SD=\sum_{x=1}^{X}\sum_{y=1}^{Y}\frac{{\left|D_{1\left(k-1\right)}\left(x,y\right)-D_{1k}\left(x,y\right)\right|}^{2}}{D_{ik}^{2}\left(t\right)}$$

The process of two-dimensional Empirical Mode Decomposition can be roughly summarized as follows.

● Input the original two-dimensional grayscale image to be processed.
● Using the domain comparison method to identify local maxima and local minima of the two-dimensional surface separately.
● Performing a planar Delaunay triangulation [28] on the local maxima and local minima found in step three, then using interpolation to smoothly construct the upper envelope surface and the lower envelope surface, and calculating their averages.
● Subtracting the mean from the input data.
● Checking if the stopping criteria are met. If they are met, proceed to the next step; if not, return to step three and continue the decomposition process.
● Using the results of step six as the n th intrinsic mode function component.
● Checking if the termination condition for the two-dimensional grayscale image is met. If it is met, output the decomposition results; otherwise, proceed to step nine.
● Removing the n th IMF component from the two-dimensional grayscale image and returning to step one, repeating the aforementioned process.

The above is the complete process of two-dimensional EMD. It involves removing the n th IMF component obtained from decomposing the original two-dimensional surface, resulting in the residual term of this decomposition.

### 2.3 Fractals and multifractals

Fractal dimension is a mathematical concept used to describe the complexity of fractal objects. Unlike traditional Euclidean geometry, where objects are described using integer dimensions, fractal objects exhibit self-similarity at different scales, leading to non-integer dimensions. Fractal dimension provides a means to quantify the characteristics of fractal objects and enables their classification and comparison. The concept of fractal geometry, introduced by mathematician Mandelbrot [29], enhances our understanding of complex and irregular structures in nature and bridges the gaps left by traditional geometric approaches.

Fractals that adhere to the definition of having Hausdorff dimension greater than the topological dimension are called regular fractals. For example, the Cantor set is a point set formed by an infinite number of points, with a topological dimension of zero. However, its Hausdorff dimension is 0.631. By applying the dimension calculation formula, the fractal dimension can be obtained as D = log2/log3 = 0.631.The box-counting method is one of the most commonly used techniques for studying multifractal surfaces. It provides a calculation approach that allows for the analysis of such surfaces.

Let A be an non-empty bounded subset of $R^{n}$ space. For any given r > 0, $N_{r}\left(A\right)$ represents the minimum number of n-dimensional cubes (boxes) with edge length r needed to cover A. If there exists a non-negative number d such that as r approaches 0, $N_{r}\left(A\right)\propto 1/r^{d}$, then d is called the box-counting dimension of A. The box dimension is d if and only if there exists a positive number k such that [15]:

(2) $$\lim_{r\to 0}\frac{N_{r}\left(A\right)}{1/r^{d}}=k$$

Furthermore, we obtain:

(3) $$d=\lim_{r\to 0}\frac{\log\;k-\log\;N_{r}\left(A\right)}{\log\;r}=-\lim_{r\to 0}\frac{\log\;N_{r}\left(A\right)}{\log\;r}$$

Multifractal analysis, also known as multi-scale fractal or complex fractal analysis [30], is an effective mathematical tool for describing the singularity structure of signals [31]. It is often used to represent the singularity probability distribution that cannot be fully described by a single global characteristic scale index. Because multifractal analysis can comprehensively describe the singularity structure of images from both local and global perspectives [32], its local characteristics can be obtained from the Hölder exponent. Meanwhile, its global characteristics can be derived from the geometric properties and probability distribution (i.e., multifractal spectrum) of the Hölder exponent. It is particularly suitable for the processing and analysis of irregular images that are difficult to model. Due to its excellent local and global properties, multifractal analysis provides a novel approach for image edge detection [33].

The box-counting method [27] was used to characterize multifractal spectra. The images can be divided into many small boxes of size l×l. Let ε=l/L(L=256) and $P_{ij}\left(\varepsilon \right)$ is the deposition probability of the QDs in the box (i,j). Thus, $P_{ij}\left(\varepsilon \right)$ is defined as:

(4) $$P_{ij}\left(\varepsilon \right)=\frac{z_{ij}}{\sum z_{ij}}$$

where $z_{ij}$ represents the average height of QDs deposition inside the box of size ε. $P_{ij}$ is the height distribution probability of the (i,j)th pixel box, it can be characterized as having multifractal properties when:

(5) $$P_{ij}\left(\varepsilon \right)∼\varepsilon^{\alpha }$$

The number of boxes of ε with the same probability is defined as $N_{\alpha }\left(\varepsilon \right)$,which has the following relationship:

(6) $$N_{\alpha }\left(\varepsilon \right)∼\varepsilon^{-f\left(\alpha \right)}$$

where α represents the singularity of the subset of probabilities, and f(α) represents the singular spectrum of the subset. In other words, f(α) is the fractal dimension of the box having the singularity of strength α. In terms of Renyi information dimension [34], the partition function $\chi_{q}\left(\varepsilon \right)$ is defined as follows:

(7) $$\chi_{q}\left(\varepsilon \right)=\sum P_{ij}{\left(\varepsilon \right)}^{q}=\varepsilon^{\tau \left(q\right)}$$

Theoretically, if the size of the box approaches zero, the generalized fractal dimension $D_{q}$ [35] can be defined as:

(8) $$D_{q}=\frac{1}{q-1}\lim_{L\to 0}\frac{\ln\sum_{i}P_{i}^{q}\left(\varepsilon \right)}{\ln\left(\varepsilon \right)}=\frac{1}{q-1}\lim\frac{\ln\;\chi_{q}\left(\varepsilon \right)}{\ln\;\varepsilon }$$

Associated with different scaling indices, q is varied in different sets.

(9) $$D_{q}=\frac{1}{q-1}\left[q\alpha \left(q\right)-c\right]$$

α(q) and $D_{q}$ also satisfy the following relationship:

(10) $$\alpha \left(q\right)=\frac{d}{dq}\left[\left(q-1\right)D_{q}\right]$$

So if f(α) is known, and the α values are also known, $D_{q}$ can be calculated. The fractal dimension f(α(q)) can be obtained by performing a Legendre transformation as follows:

(11) $$\alpha =\frac{d}{dq}\left[\tau \left(q\right)\right]$$

(12) f(α)=αq−τ(q)

In practical calculations, the value of q is bounded and cannot be infinite. When we analysis the multifractal spectrum, a series of parameters are available for us to discover the characteristics of different images. The width of the multifractal spectra is Δα, and the difference of the fractal dimensions of the maximum probability subset $\alpha_{\min}$, and the minimum one $\alpha_{\max}$ is Δf:

(13) $$\Delta f=f\left(\alpha_{\min}\right)-f\left(\alpha_{\max}\right)$$

Here is a pseudo code scripting the implementation in order to make this a repeatable research.

We can extract 9 features from it for subsequent classification and feature extraction. These features can be illustrated in Fig. 4, Fig. 5 and listed as follows.

1. The maximum value of α ($\alpha_{\max}$) in the multifractal spectrum,
2. The multifractal spectrum at the αmax ($f\left(\alpha_{\max}\right)$),
3. The minimum value of α ($\alpha_{\min}$) in the multifractal spectrum,
4. The multifractal spectrum at the αmin ($f\left(\alpha_{\min}\right)$),
5. The α value at the maximum of the singularity spectrum curve ($\alpha_{0}$),
6. The width of the multifractal spectrum curve (Δα),
7. The box-counting dimension ($D_{0}$),
8. The Information dimension ($D_{1}$),
9. The correlation dimension ($D_{2}$).

**Fig. 3:**
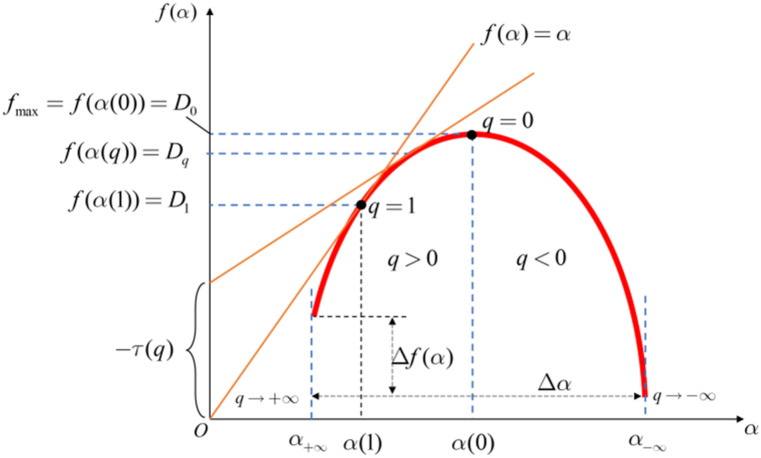
A schematic of the multifractal spectrum and its related parameters.

**Fig. 4:**
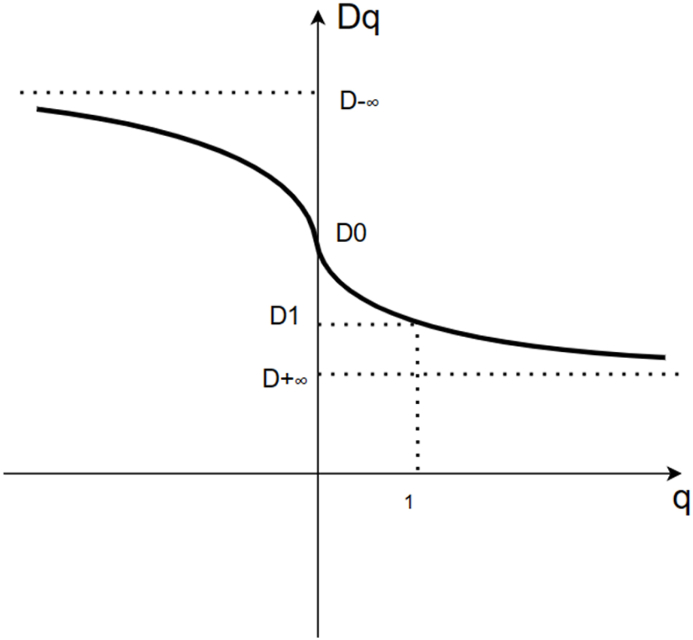
The multifractal generalized dimension.

**Fig. 5:**
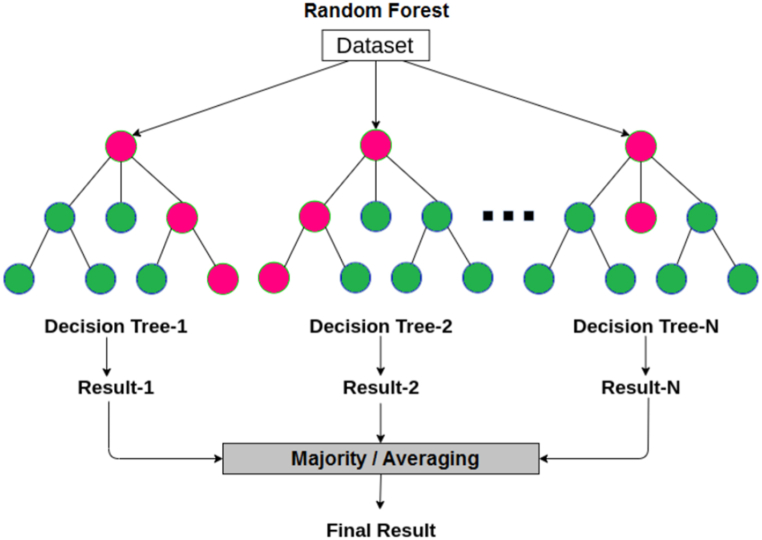
The random forest algorithm.

### 2.4 K-means method for retina images classification and correlation analysis

Based on the experimental results mentioned above, we selected two dimensions as indicators for K-means clustering. K-means is a simple and easy-to-understand clustering algorithm. It is straightforward to implement and does not require complex parameter tuning. Its results are easy to interpret. Each data point is assigned to a specific cluster, providing meaningful insights and analysis. Additionally, K-means is relatively robust to noise and outliers, especially when using appropriate distance metrics or preprocessing techniques such as feature scaling or outlier removal. We also conducted Spearman correlation analysis on various fractal parameters. Spearman correlation analysis is a statistical method used to assess the strength and direction of the monotonic relationship between two variables. It is a nonparametric measure of correlation that is based on the ranks of the data rather than the actual values. Through this method, we can determine which factors have an impact on the classification results.

### 2.5 Random forest algorithm

Random Forest is an ensemble learning method used for solving classification and regression problems. It is based on the ensemble of decision trees and utilizes random feature selection and voting mechanism to enhance the accuracy and robustness of the model. To initiate the training of the Random Forest algorithm, three parameters need to be adjusted in order to function as a classifier. These parameters can be summarized as follows: (1) the number of trees to be used, (2) the number of nodes within each tree, and (3) the number of features to be sampled. This is illustrated in Fig. 3.

In almost all classification systems, hundreds or thousands of features are used to obtain accurate results. However, not all extracted features are equally important or have a strong influence in the classification process. Therefore, it is necessary to create a classification model that includes only the most relevant features, which is referred to as "feature selection".

## 3 Experimental results

The tools used in this experiment for performing two-dimensional empirical mode decomposition and multifractal analysis on the image were Matlab 2022a. The subsequent feature importance analysis was performed using Python 3.8.5. The model proposed in this paper is trained and tested on a PC with 13th Gen Intel(R) Core(TM)i9-13900HX CPU,16 GB memory and NVIDIA GeForce RTX 4060 LapTop graphics card. In this experiment, two datasets were used: the Drive dataset and an additional dataset. The Drive dataset consists of a total of 40 JPEG format color fundus images, including 7 cases with abnormal pathologies. The images were captured using a Canon CR5 non-mydriatic 3CCD camera with a field of view (FOV) equal to 45°. Each image has a resolution of 584 x 565 pixels, and each color channel has 8 bits. Another dataset is an open standard diabetic retinopathy database, comprising 35,126 retina scan images. The size of these images is resized to 224x224 pixels. Among them, the diseased retinal images are categorized into four different severity levels: mild, moderate, severe, and proliferative DR.

**Fig. 6:**
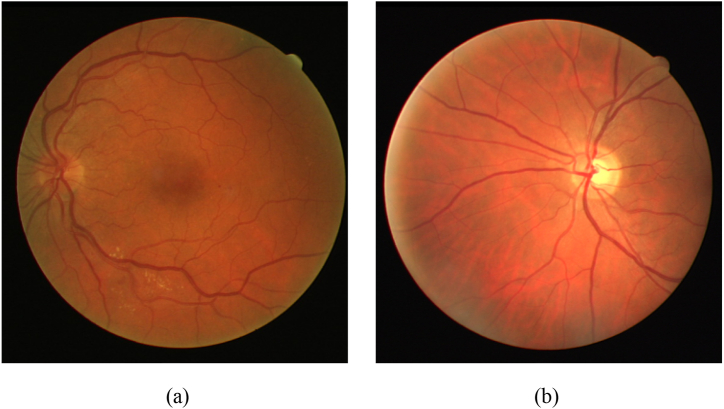
Retina images of lesion (a) and healthy (b).

This section discusses the results of classifying pathological retinal images and normal retinal images, fractal parameters, two-dimensional HHT, and multifractal analysis of segmented regions. A random forest model was used to analyze the importance of the 9 extracted features. As shown in Fig. 6, we selected retinal images from a group of diabetic patients and healthy individuals.

### 3.1 2D HHT process

**Fig. 7:**
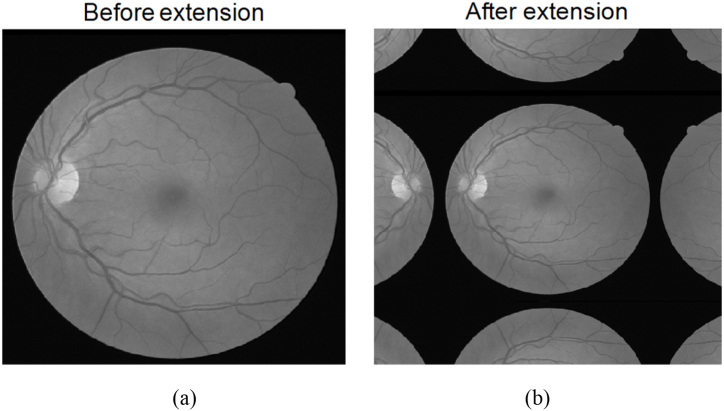
Original image (a) and mirror extended image (b).

First, we transferred the original image to a two dimensional gray image. And then we utilize the method described in the first section, the extrema point selection method for two-dimensional grayscale images is employed. The eight-neighborhood approach is used to select the local maximum and minimum points of the two-dimensional grayscale image. In one-dimensional empirical mode decomposition, a point in a function can only be either a local maximum or a local minimum. For local maximum points, they do not contribute to the construction of the lower envelope line. Similarly, for local minimum points, they do not affect the construction of the upper envelope line. This significantly affects the accuracy of the empirical mode decomposition results, and with each iteration, this effect becomes more pronounced. The boundary effect also exists in two-dimensional empirical mode decomposition because it involves constructing upper and lower envelope surfaces using the local maximum and minimum points of the image. To address the boundary effect in two-dimensional empirical mode decomposition, the image is subjected to mirror extension processing, as shown in Fig. 7.

**Fig. 8:**
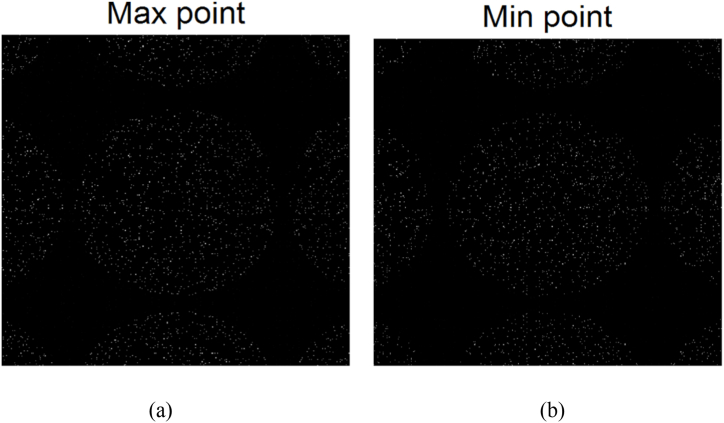
Points of local maximum (a) and local minimum (b).

In Fig. 3, the bright white dots in Fig. 3(a) represent the positions of local maximum points, while the bright white dots in Fig. 3(b) represent the positions of local minimum points. It can be observed that the eight-neighborhood comparison method effectively identifies the locations of the maximum and minimum points in the image, as shown in Fig. 8.

**Fig. 9:**
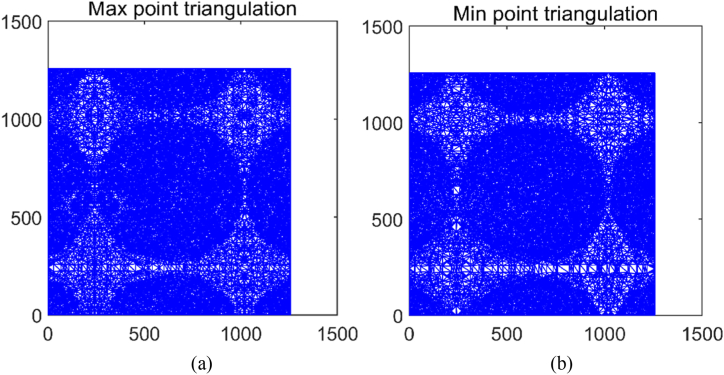
Delaunay triangulation for maximum (a) and minimum values (b).

Next is the part of surface fitting. In one-dimensional EMD, the core idea is to continuously extract the average envelope of the signal from the original signal. In two-dimensional Empirical Mode Decomposition, the concept is to continuously remove the average envelope surface of the signal from the original signal. Therefore, the construction of the envelope surface is crucial for the decomposition and analysis of the two-dimensional EMD. We use Delaunay triangulation and Cubic interpolation to find the maximum and minimum value envelope surfaces of two-dimensional grayscale images. By establishing a large triangle or polygon that encompasses all the data, it is then divided into two original triangles. Data points are inserted and connected to the three vertices of the triangle that contains them, forming new triangles. This process is repeated until a stopping criterion is met. The triangulation is illustrated in Fig. 9.

**Fig. 10:**
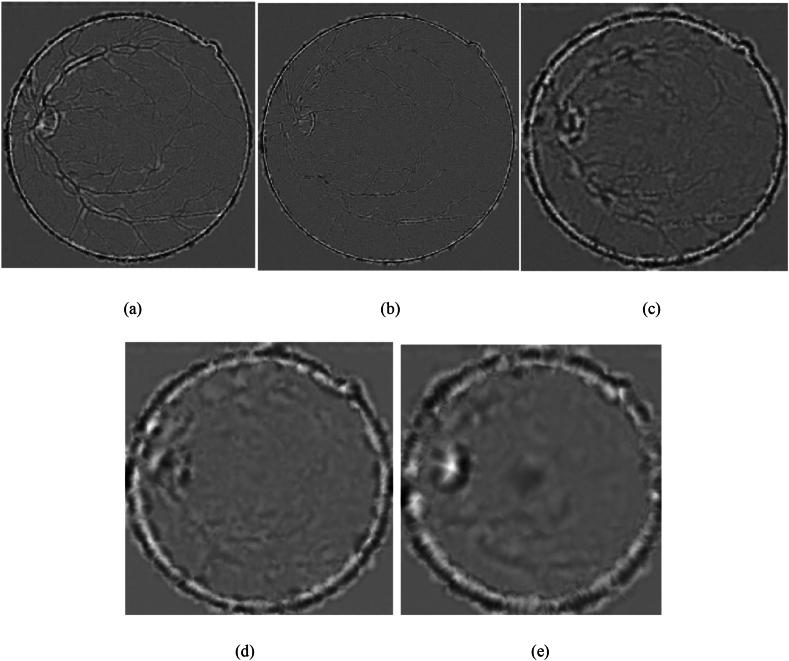
Five IMFs of one retina image. The first intrinsic mode function component (a) displays some detailed information about the image. The second intrinsic mode function component (b) is the detailed information extracted after removing the first component, and so on.

**Fig. 11:**
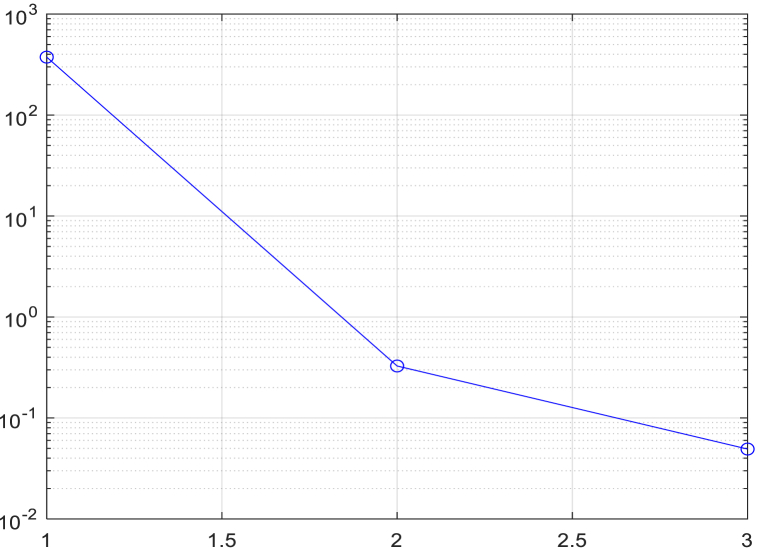
Changing process of SD.

Using the stopping criterion as a guideline, iterative decomposition is performed until SD = 0.2, resulting in five intrinsic mode components of the original image, as shown in Fig. 10. Fig. 11 illustrates the process of SD changing with the increasing number of iterations.

IMF 1 typically contains the highest frequency components of the signal, corresponding to details and noise. It exhibits relatively fast oscillatory patterns with shorter time scales. As the number of IMF increases, the frequency components gradually decrease, and the time scale gradually increases. Therefore, higher-numbered IMF components typically correspond to lower-frequency oscillatory patterns. As we progress further, the extracted intrinsic mode function components become more blurred. This is because the bidimensional empirical mode decomposition is a process of continuously extracting high-frequency components from the signal. Therefore, the bidimensional empirical mode decomposition is capable of effectively extracting the detailed information of the image. This sets the stage for subsequent multi-scale analysis and further processing.

### 3.2 Multifractal analysis process

**Fig. 12:**
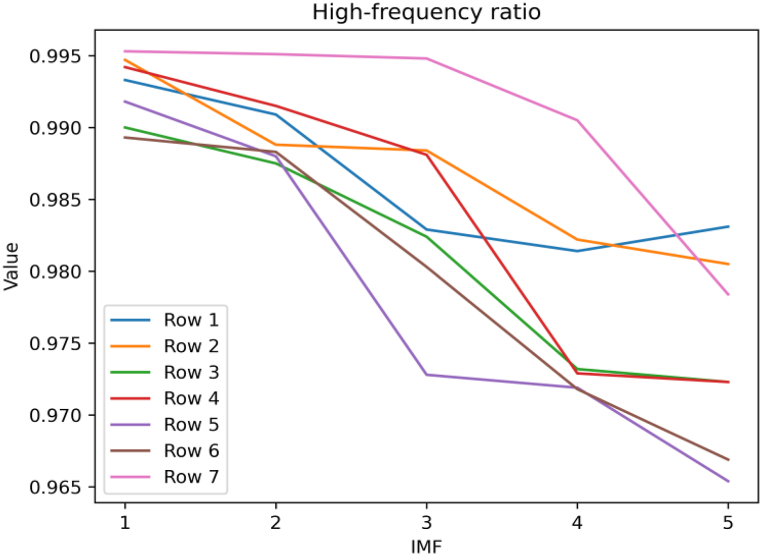
High-frequency ratio of IMF1 to IMF5.

We perform multifractal analysis on the image transformed through the 2D HHT using the box-counting method. However, before that, it is worth discussing how to select IMF components for multiscale analysis and further processing. We first selected a subset of the 5 IMF components obtained from EMD decomposition for Fourier transform and calculated the power spectrum in the frequency domain. We then calculated the percentage of energy contributed by the high-frequency components.The results are as shown in Fig. 12.

**Fig. 13:**
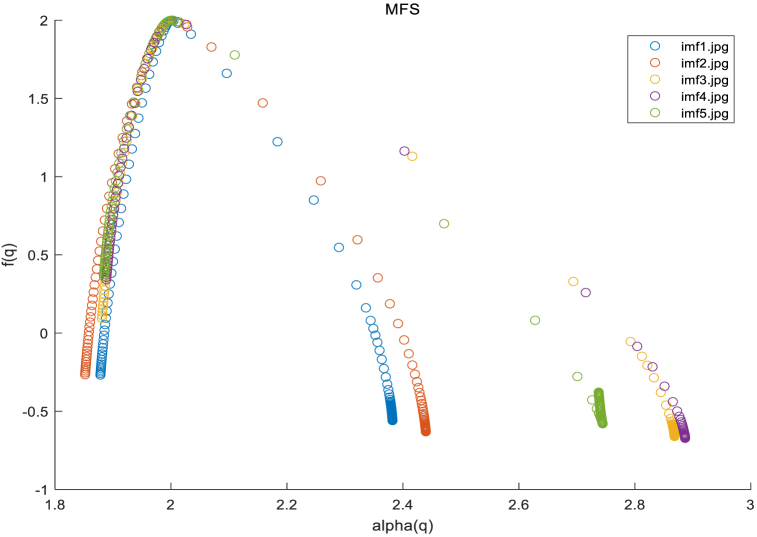
MFS of five imfs.

The experimental results validate the earlier claim that IMF 1 typically contains the highest frequency component of the signal corresponding to details and noise. Furthermore, we conducted multiple experiments and found that the IMF1 component, compared to the other components, better demonstrates fractal characteristics. Here is an example of a retina, Fig. 13.

In conclusion, among the 5 IMF components, IMF1 represents the high frequency components of the data and contains the detailed information of the image. It captures finer and clearer image lines compared to the low-frequency contour features of IMF5. Hence, it can better reflect the differences in texture structures. Therefore, the fractal analysis of the grayscale image primarily relies on the fractal spectrum derived from IMF1.

**Fig. 14:**
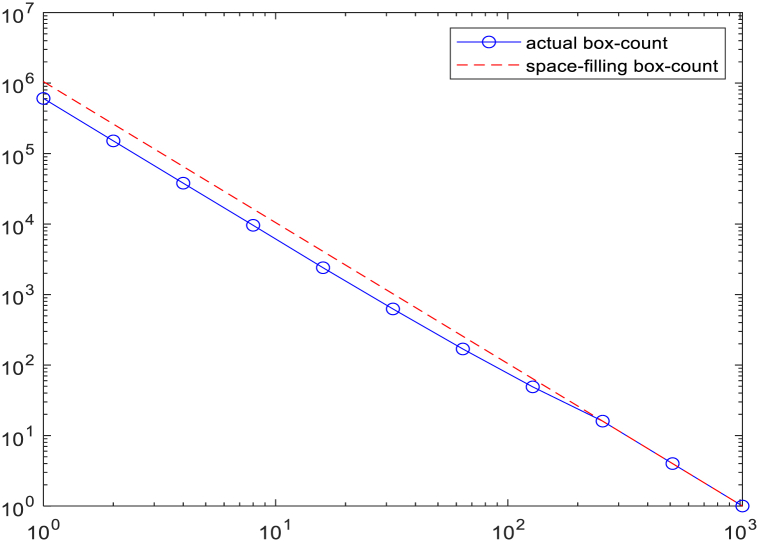
The cocmparison between actual box-count and space-filling boxcount of one retina.

This part we uses the box counting method to calculate the FD of the retina. This method has been used in many studies due to its robustness, accuracy, and ease of implementation. The FD can be derived from the following power-law relationship：

(14) $$\ln\left(N\left(r\right)\right)=FD\cdot \ln\left(r^{-1}\right)+C$$

where C is a constant. The slope of the line can be used to estimate the FD value, Fig. 14.

**Fig. 15:**
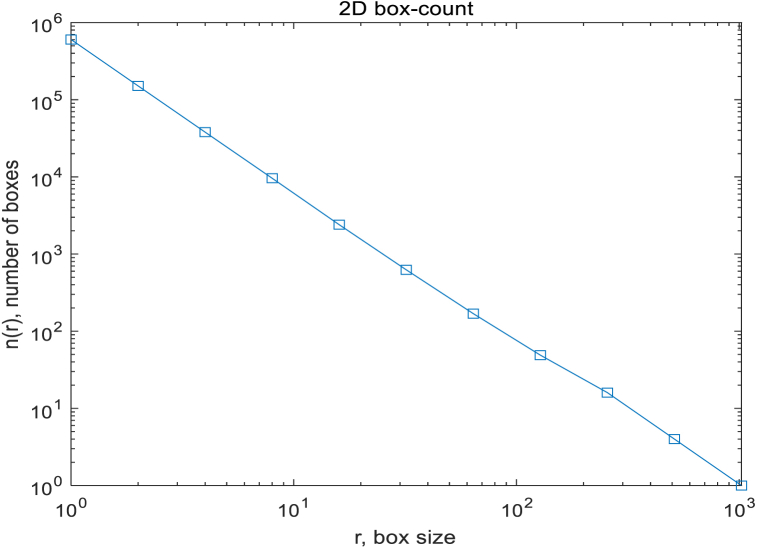
Relationship between the number of boxes and their sizes of one retina.

**Fig. 16:**
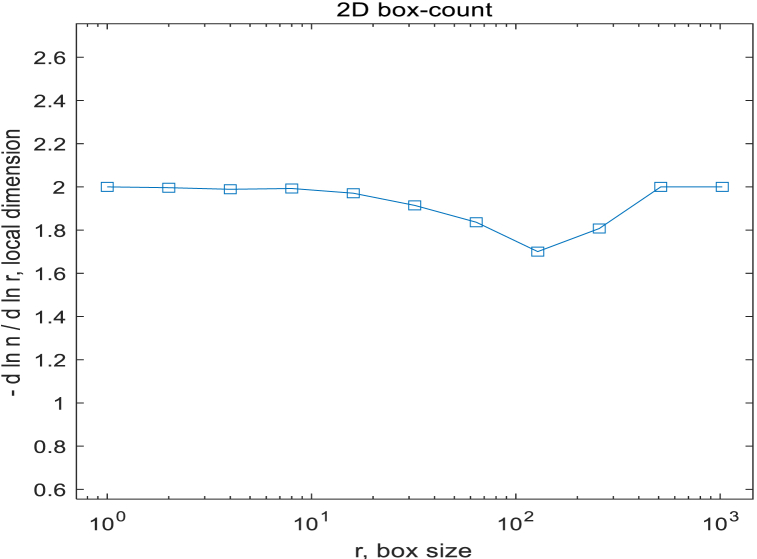
Relationship between the FD and the box sizes of one retina.

The red dotted line shows the scaling N(R)=R^-2 for comparision, expected for a space-filling 2D image. The discrepancy between the two curves indicates a possible fractal behaviour. Due to the fact that retinal images are not purely fractal, it is necessary to select an appropriate range of box sizes. In this study, a complete slope analysis was conducted, evaluating all possible values for the number of boxes of different sizes to determine the appropriate range of box sizes, as shown in Fig. 15 and Fig. 16.

**Fig. 17:**
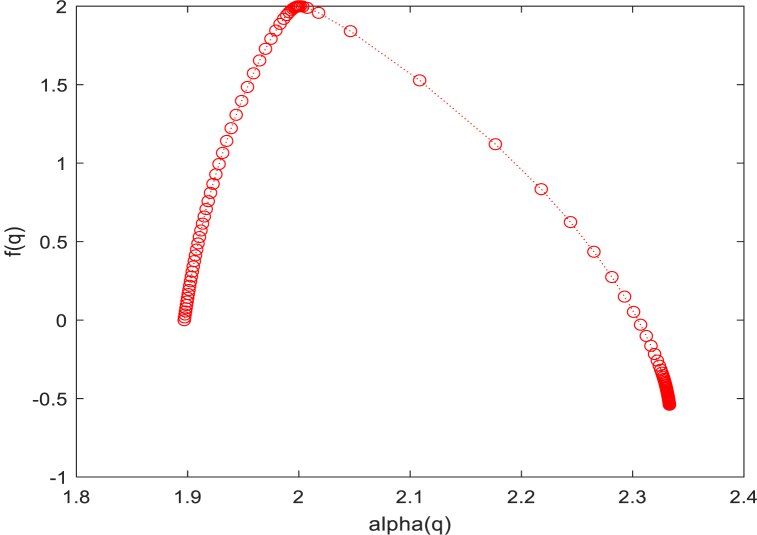
Multifractal spectrum of one patient's retina.

**Fig. 18:**
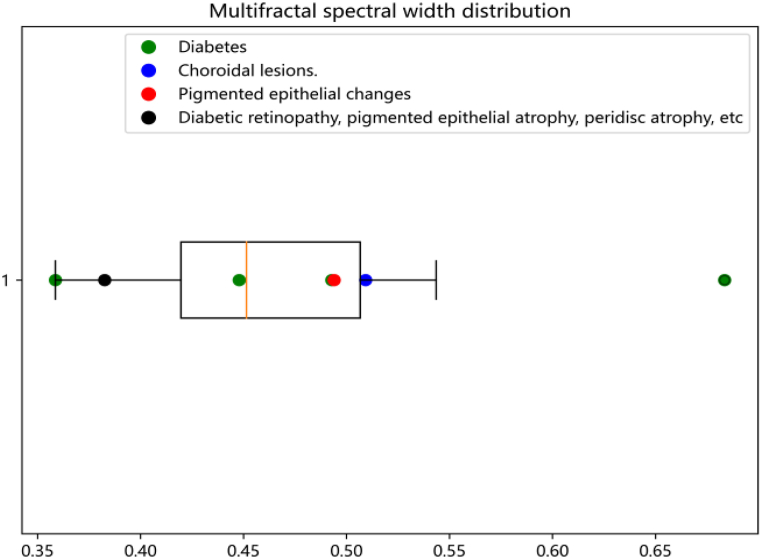
Distribution of Multifractal spectrum widths.

Then, we plotted the multifractal spectra of 40 retinal images from the Drive dataset. In multifractal spectra, the values of the multifractal spectrum f(α) corresponding to larger singular value indices α reflect the features within the low probability measure regions of the image. On the other hand, the values of the multifractal spectrum f(α) corresponding to larger singular value indices α capture the features within the high probability measure regions of the image. In the multifractal spectrum in Fig. 17, the width represents the difference between the maximum and minimum probabilities. It reflects the degree of unevenness in the probability measure distribution of the entire fractal structure. It describes the physical characteristics of the fractal structure in different regions or levels. A larger width indicates more pronounced multi-fractal features in the image, further emphasizing the ability of the multi-fractal spectrum to represent the structural characteristics of the image. So, we recorded the multifractal spectrum widths for each image, and present the distribution of the data using a box plot, Fig. 18.

From the box plot, we can observe that there are significant outliers in the multifractal spectrum width of retinal images in diabetic patients. So we selected more images to find common characteristics. We chose an open standard diabetic retinopathy database, comprising 35,126 retina scan images. The size of these images is resized to 224x224 pixels. Among them, the diseased retinal images are categorized into four different severity levels: mild, moderate, severe, and proliferative DR. For more information about the diabetic retinopathy database, please refer to “https://www.kaggle.com/datasets/sovitrath/diabetic-retinopathy-2015-data-colored-resized”. We chose 60 images with 20 healthy and 40 lesion retina images and plotted the multifractal spectra.

**Fig. 19:**
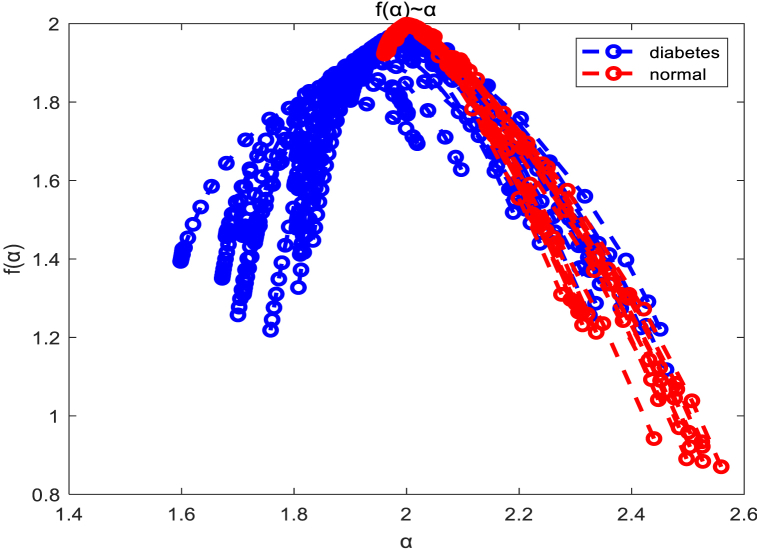
Multifractal Spectrum of diabetes and normal individuals.

Fig. 19 displays the results of multifractal spectrum calculations using the texture data from retinal images of both healthy individuals and patients with diabetes, based on the aforementioned algorithm. It can be observed that, compared to healthy individuals, the multifractal spectrum of diabetic patients' retinal images exhibits a longer right tail, indicating that the periodic intensity of retinal texture images is generally lower in healthy individuals than in diabetic patients [36].

**Fig. 20:**
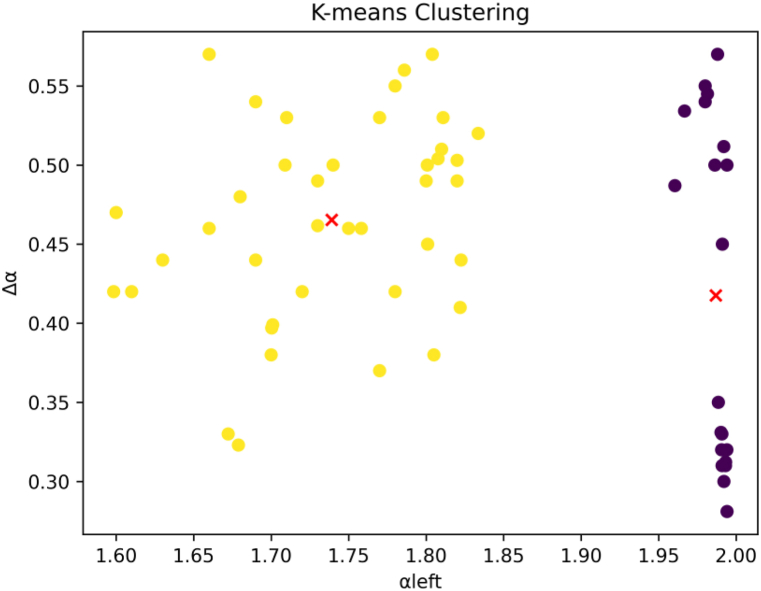
Retinal k-means clustering.

Next, we take αmin as the first dimension and the multifractal spectrum width Δα as the second dimension, and plot a series of two-dimensional points. We attempt to differentiate them using k-means clustering. The results are as follows. Yellow points represent diabetic patients, and the black ones represent healthy individuals, Fig. 20. The clustering has achieved satisfactory results.

We further selected parameters such as multifractal spectrum width, fractal dimension (FD), etc., for Spearman correlation analysis to investigate the degree of correlation between these parameters and the disease condition.

**Fig. 21:**
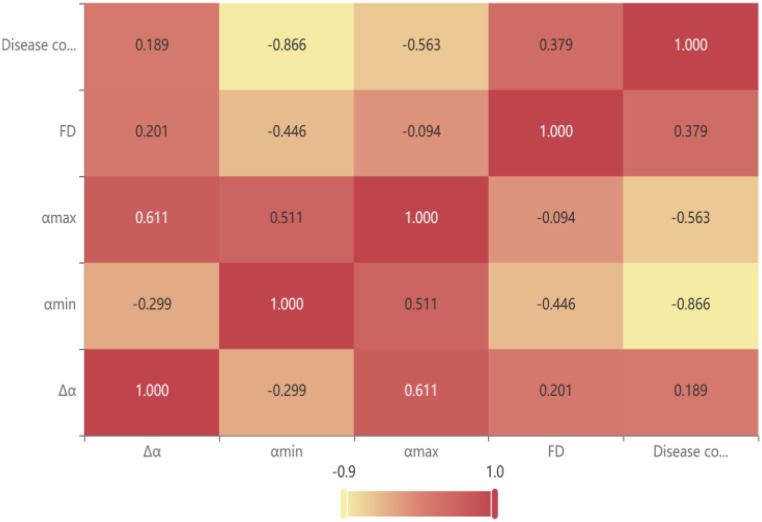
Spearman's correlation analysis between parameters.

Fig. 21 shows a heatmap representation of the correlation coefficients, where the values are primarily represented by varying shades of color to indicate their magnitude. Among them, αmin and αmax exhibit a higher negative correlation with the disease condition, while FD is moderately correlated, and Δα has the weakest correlation. These results provide valuable parameter selection recommendations for future research and suggest that the proposed method may be a promising clinical system.

### 3.3 Feature importance analysis

**Fig. 22:**
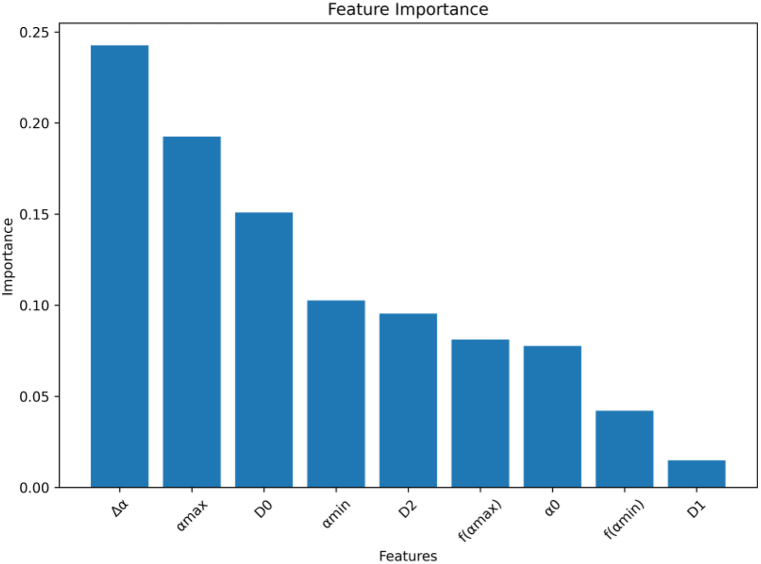
The features importance using RF algorithm.

**Fig. 23:**
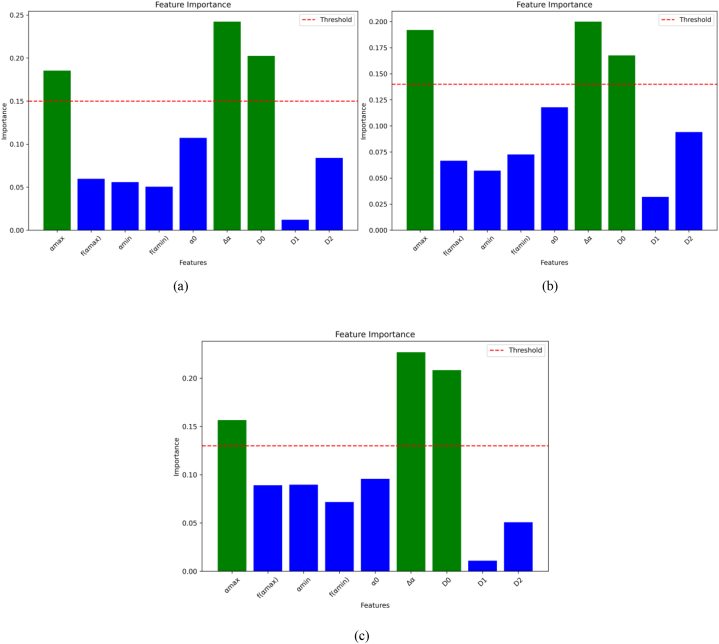
Ranking of the feature importance provided by RF. The performance of the model using different thresholds, which is 0.15 (a), 0.14 (b), and 0.13 (c), respectively.

According to the proposed method, 9 features have been extracted, which describe the retina. Sample of some extracted features data are shown in Table 2. To reduce the number of features used, the RF algorithm is employed to eliminate less relevant features. Table 1 summarizes partial patients' retinal images with extracted feature values. According to Fig. 22, the important features can be concluded as Δα, αmax, D0. Fig. 23 shows the ranking of the feature importance provided by RF. We evaluate the performance of the model using different thresholds, namely 0.15, 0.14, and 0.13, respectively. The blue parts (features) are discarded as being under the threshold value. Performing a model evaluation using multiple thresholds, the optimum threshold value can be chosen as 0.15.

**Table 1:** Pseudo code of box-counting algorithm.

Algorithm 1: Box-counting algorithm
Input: dataset D = {(x,y), (x,y),(x,y)}
Output: dimensions
1:dimensions = [] # Used to store box count dimensions at each scale
2: **for** box_size in box_sizes:
3: box_count = 0
4: box_dimension = 0
5: # Divide bounding boxes and count the number of boxes
6： **for** i from 0 to N step box_size:
7： **for** j from 0 to N step box_size:
8： **if** has_point(fractal, i, j, box_size):
9： box_count + = 1
10： # Calculate box dimensionality
11： **if** box_size >0 and box_count >0:
12： box_dimension = log(box_count)/log(box_size)
13： dimensions.append(box_dimension)
14： return dimensions

**Table 2:** Sample of some extracted features data.

Id	αmax	f(αmax)	αmin	f(αmin)	α0	Δα	D0	D1	D2
Mild1	2.2994	−0.5903	1.8597	−0.3347	2.0003	0.4397	2.0002	1.9997	1.9994
Mild2	2.4443	−0.5770	1.8814	0.0051	1.9932	0.5629	2.0	1.9765	1.9489
Mild3	2.3330	−0.5395	1.8967	0	2.0135	0.4633	2.0001	1.9435	1.9177
Moderate1	2.3087	−0.4439	1.8774	−0.2193	2.0018	0.4313	1.9987	1.9952	1.9660
Moderate2	2.2401	−0.4513	1.8761	−0.2835	2.0077	0.3640	1.99	1.9631	1.9225
Moderate3	2.1883	−0.5156	1.9041	−0.094	2.0031	0.2842	1.9959	1.9732	1.973
Severe1	2.2365	−0.3434	1.9182	0.2338	2.012	0.3183	1.9975	1.9953	1.9833
Severe2	2.1927	−0.4873	1.8585	−0.5373	2.0014	0.3342	2.00	1.9876	1.977
Severe3	2.5173	−0.6799	1.8756	−0.3662	2.0079	0.6417	1.9974	1.9732	1.971

## 4 Discussions

In this study, we validated the effectiveness and robustness of our proposed model, which consists of 2D EMD and multifractal analysis, using a dataset of 60 fundus images from the Standard Diabetic Retinopathy Database. Many existing methods for retinal image classification have achieved high classification accuracy. However, some of these models are complex and involve multiple steps, making the implementation of the classification algorithm challenging. It is worth mentioning that the proposed classification system in this paper is straightforward to implement. On the other hand, the system achieves satisfactory classification accuracy. Therefore, it can be seen that it will contribute to the classification of mutations in pathological patterns within biological tissues based on multifractal characteristics. The proposed system achieved classification accuracy, sensitivity, and specificity of 96.24%, 95.56%, and 95.38% respectively. Table 3 shows the comparison with the results published in the related work of others.

**Table 3:** The comparison of achieved results with state-of-the-arts.

Task Methods Subjects number Performance
Prasad [37] Haar wavelet 89 Accuracy:97.8% Sensitivity:97.5% Specificity:97.75%
Omar [38] Multiscale LBP texture 130 Accuracy:98.68% Sensitivity:94.81% Specificity: 96.73%
Jian Wang [13] 2D MF-DFA-LSSVM 130 Accuracy:99.01% Sensitivity:99.03% Specificity: 97.73%
Koh [39] Continuous wavelet 910 Accuracy:92.48% Sensitivity:89.37% Specificity:95.58%
Acharya [40] Features from Gabor 510 Accuracy:93.10% Sensitivity:89.75% Specificity:96.20%
Stevenson [41] Artificial intelligence algorithms 4435 Accuracy:89.00% Sensitivity:75.00% Specificity:89.00%
Maheshwari [42] VMD and entropy 488 Accuracy:95.19% Sensitivity:93.62% Specificity:96.71%
Giraddi [43] Haar wavelet and First order statistical features 130 Accuracy:85% Sensitivity:87% Specificity:80%
Ours 2D EMD and Multifractal 60 Accuracy:96.24% Sensitivity:95.56%
Specificity:95.38%

## 5 Conclusions

Diabetic retinopathy is one of the most common complications in patients with diabetes. High blood sugar levels can damage the microvasculature in the retina, leading to vascular abnormalities and the formation of abnormal new blood vessels. The main novelty of this study lies in extending the one-dimensional Hilbert-Huang Transform to two dimensions and applying it to pattern recognition in two-dimensional retinal lesion images. Additionally, it combines multifractal spectrum analysis with machine learning to classify the texture of retinal images, demonstrating the impact of diseases on the retinal microstructure. Furthermore, the feasibility of lesion classification is demonstrated. The presented results indicate that diabetic patients' retinal images exhibit stronger fractal features compared to retinal images of healthy individuals. The main contributions of this paper are as follows: (1) extending the one-dimensional empirical mode decomposition to two dimensions; (2) applying empirical mode decomposition to pattern recognition in two-dimensional retinal lesion images; (3) quantifying the complexity of retinal images through multifractal analysis of Hilbert Huang transform images; (4) extracting fractal features of image to distinguish the retina of diabetes patients and normal people; (5) using a random forest model for feature importance analysis to separate potentially important features. The 2D EMD + Multifractal model's effectiveness in classifying diseases is validated from various perspectives, including accuracy, sensitivity and specificity.

Currently, an increasing number of studies indicate that many tissues and organs in the human body exhibit specific fractal behavior. Therefore, fractal analysis, as a powerful tool for quantifying the complexity of irregular images, will be a valuable tool in future medical image diagnostics. However, much of the current research on fractals has been limited to natural images or other two-dimensional medical images, with limited application to the study of three-dimensional object morphology. The quality of the dataset images is also a major limitation of the proposed method.

## CRediT authorship contribution statement

**Lei Yang:** Conceptualization. **Minxuan Zhang:** Writing – original draft, Visualization, Investigation, Formal analysis. **Jing Cheng:** Formal analysis. **Tiegang Zhang:** Formal analysis. **Feng Lu:** Formal analysis.

## Declaration of competing interest

The authors declare that they have no known competing financial interests or personal relationships that could have appeared to influence the work reported in this paper.
